# Comparative dynamics of mixed convection heat transfer under thermal radiation effect with porous medium flow over dual stretched surface

**DOI:** 10.1038/s41598-023-40040-9

**Published:** 2023-08-07

**Authors:** Mohammad Mahtab Alam, Mubashar Arshad, Fahad M. Alharbi, Ali Hassan, Qusain Haider, Laila A. Al-Essa, Sayed M. Eldin, Abdulkafi Mohammed Saeed, Ahmed M. Galal

**Affiliations:** 1 Department of Basic Medical Sciences, College of Applied Medical Science, King Khalid University, 61421 Abha, Saudi Arabia; 2 Department of Mathematics, University of Gujrat, Gujrat, 50700 Pakistan; 3 Department of Mathematics, Al-Qunfudah University College, Umm Al-Qura University, Mecca, Saudi Arabia; 4 Department of Mathematical Sciences, College of Science, Princess Nourah bint Abdulrahman University, P.O. Box 84428, 11671 Riyadh, Saudi Arabia; 5 Center of Research, Faculty of Engineering, Future University in Egypt, New Cairo, 11835 Egypt; 6 Department of Mathematics, College of Science, Qassim University, 51452 Buraydah, Saudi Arabia; 7 Department of Mechanical Engineering, College of Engineering in Wadi Alddawasir, Prince Sattam bin Abdulaziz University, Al-Kharj, Saudi Arabia; 8 Production Engineering and Mechanical Design Department, Faculty of Engineering, Mansoura University, Mansoura, P.O 35516 Egypt

**Keywords:** Applied mathematics, Fluid dynamics

## Abstract

Due to enhanced heat transfer rate, the nanofluid and hybrid nanofluids have significant industrial uses. The principal objective of this exploration is to investigate how thermal radiation influences the velocity and temperature profile. A water-based rotational nanofluid flow with constant angular speed ${\Omega }$ is considered for this comparative study. A similarity conversion is applied to change the appearing equations into ODEs. Three different nanoparticles i.e., copper, aluminum, and titanium oxide are used to prepare different nanofluids for comparison. The numerical and graphical outputs are gained by employing the bvp-4c procedure in MATLAB. The results for different constraints are represented through graphs and tables. Higher heat transmission rate and minimized skin friction are noted for triple nanoparticle nanofluid. Skin coefficients in the x-direction and y-direction have reduced by 50% in trihybrid nanofluid by keeping mixed convection levels between the range $3 <\upepsilon \le 11$. The heat transmission coefficient with raising the levels of thermal radiation between $0.5 < {\uppi } \le 0.9$ and Prandlt number $7 < {\mathrm{Pr}} \le 11$ has shown a 60% increase.

## Introduction

Incompressible fluid movement above a extending surface has numerous applications in the industrial field like aerodynamics, extrusion of plastic sheets, continuous metallic plate extrusion, artificial fibers, and stretching of plastic films. In each of these cases, the amount of heat transfer at the stretchy surface influences the effectiveness of the final form of the product. To learn the fundamentals of these processes, it is essential to examine dynamics and thermal transmission within a fluid above a continuously extending sheet/surface. Sakiadis^1^ examined boundary layer (BL) behavior over continuous solid surfaces elaborating on the two-dimensional axisymmetric movement. Erickson et al.^2^ discussed the injection/suction influence on heat and mass transmission above a moving flat plate. Crane^3^ explored the flow past the stretchable surface and Chen and Stroble^4^ scrutinized the buoyancy influence on a flat plate. Turkyilmazoglu^5^ explored the stretching wall in magneto-hydrodynamic rotating flow frames. Takhar and Nath^6^ explored three-dimensional movement over the stretched surface. Several investigators have examined stretching surfaces for heat and mass transfer under different under-applying forces^7–12^.

The dispersion of NPs in a host fluid is known as nanofluid (NF). Similarly, the addition of one or more nanoparticles in NF is termed as a hybrid nanofluid (HNF). Susrutha et al.^13^ elaborated that the NPs bond to and through the other molecules which govern different properties like heat transfer rate and electrical conductivity. Phule et al.^14^ studied the shape and size of $Ag$-NPs and $Ag$-$PVF_{2}$ using the electron microscope at high voltage. Behra and Ram^15–19^ investigated the mechanism of solubilizing, and stabilization in the form of nanofluid in an alcoholic medium, and variations in optical properties in fullerene $C_{60}$ with the existence of poly (vinyl pyrrolidone) molecules in water. Singh and Ram^20^ magnetic NF’s synthetization and their applications.

Stretchable surfaces in fluid environments are known to produce significant features of industrial and technological value. With the aid of a cutting-edge spectral relaxation technique, Ghasemi et al.^21^ investigated NF flow over the stretched surface with radiation and magnetic field influence. They observed that high magnetic field intensities have a significant impact on temperature profile. Arshad and Hassan^22^ explored HNF between rotating systems. Nasir and Hassan et al.^23–25^ discussed the flow of water and ethylene glycol magnetized NP over a flexible surface. Additionally, elaborated on the flow of different NFs with an energy source and entropy analysis on the transportation of MHD advanced NF embedded in the porous surface.

Due to the intrinsic rotation of the fluid particles and their tendency to ascend with fluid motion, fluid rotation occurs in nature. Researchers investigated rotational fluid paired with different properties because of engineering and industrial applications. Mehmood and Ali^26^ have investigated the impact of MHD in a revolving system of overstretched and porous media. Hussain et al.^27^ examined the rotating nanofluid flow over the stretchable surface by considering the magnetic effect. Shah and Ullah^28^ considered forced convection for ferrofluid treatment with the insertion of the electric field. Dadheech et al.^29,30^ explored the copper oxide and silver with ethylene glycol as host fluid with porous medium effect and modified NF flow above non-linear stretching surface with MHD effect. Shah et al.^31^ computationally studied engine oil base HNF with variable heat transfer.

The thermal radiation impact on the free convection flow is important in many engineering applications, such as in advanced types of power plants for nuclear rockets, reentry vehicles, high-speed flights, and procedures involving high temperatures. Sheikholeslami et al.^32^ explored radiation impact on MHD NF utilizing two-phase simulation with heat transfer. Safdar et al.^33^ examined Maxwell NF using the Buongiorno model with thermal radiation. Micro-organism profiles diminish under higher bioconvection levels they remarked. Asghar et al.^34^ presented the mixed convection HNF with slip conditions and heat generation/absorption. Jawad et al.^35–37^ studied the bioconvection and thermal radiation effect of Williamson and Casson NF with micro-organism migration over a stretching surface. They discovered that migration of micro-organisms declines with increment in bio convection and Peclet number. Similarly, numerous researchers^38–41^ have explored different NF's flow over stretchable surfaces considering diverse assumptions. Tang et al.^42^ presented a computational study on magnetized gold-blood NF flow and heat transmission. Farooq et al.^43^ employed the Cattaneo-Christov model with the bio-convectional flow of Williamson fluid under the thermal radiation effect over the slandering cylinder.

In the above-conducted literature review, it is demonstrated that the comparison between the three different types of NFs has not been yet investigated. Hussain^8^ discussed the effects of radiation and magnetization on water-based NFs, and Hassan^10^ elaborated on linear and nonlinear radiation effects on distinct-based HNFs. Adnan and Jawad et al.^36^ examined the Arrhenius energy and heat transport activates effect on gyrotactic microorganisms with Nield boundary conditions. Arshad and Hassan^22^ discussed the HNFs using different nanoparticles. The objective of the present research is to comparatively discuss the three distinct types of water-based nanofluids above dual stretching surface inserted in a porous medium, namely, NF $Cu/H_{2} O$; HNF $Cu{-}Al_{2} O_{3} /H_{2} O$ and trihybrid nanofluid (THNF) $Cu{-}Al_{2} O_{3}{-}TiO_{2} /H_{2} O$ for heat transfer rate. The novelty of this study is that we have incorporated the Rosseland thermal radiation approximation to investigate the mixed convection in rotating nanofluids flow. Turkyilmazoglu^5^ used the spectral Chebyshev collocation method, Shafiq^18^ employed the optimal homotopy method, Hayat^44^ studied heat transfer with the homotopy analysis method and in this study we use the BVP-4c technique to examine the comparative flow of different NFs with radiation effect over dual stretching. Related researches^45–49^ can be studied. The governing equations are transformed into the ordinary differential equation by employing a similarity transformation and tackled a MATLAB by applying the boundary value problem method (BVP-4c). The outcomes of the present study are described through graphs and tables for different parameters. The main research questions of the present research are designed as follows:

1. How different parameters change the temperature and velocity profiles?
2. How primary and secondary velocity is effected by mixed convection and porous medium parameter.
3. Increase in Prandtl number and radiation parameter how effects both profiles?
4. Which type of NF, HNF, or THNF shows the maximum heat transfer rate?

## Governing equations

The basic flow governing equalities for viscid incompressible liquid over the permeable surface in the existence of Rosseland radiation are:

*Continuity equation*

1 $$\nabla \cdot V = 0.$$

*Momentum equation*

2 $$\rho \left[ { V_{t} + \left( {V \cdot \nabla } \right)V} \right] = - \nabla P + \rho g + \mu \nabla^{2} V - \left[ {\frac{\mu }{K1}} \right]V.$$

*Energy equation*

3 $$\rho C_{p} \left[ {\frac{\partial T}{{\partial t}} + \left( {V \cdot \nabla } \right)T} \right] = k _{f} \nabla^{2} T - \left[ {\frac{\partial }{\partial z } qr } \right].$$

## Formulation of problem

**Figure 1:**
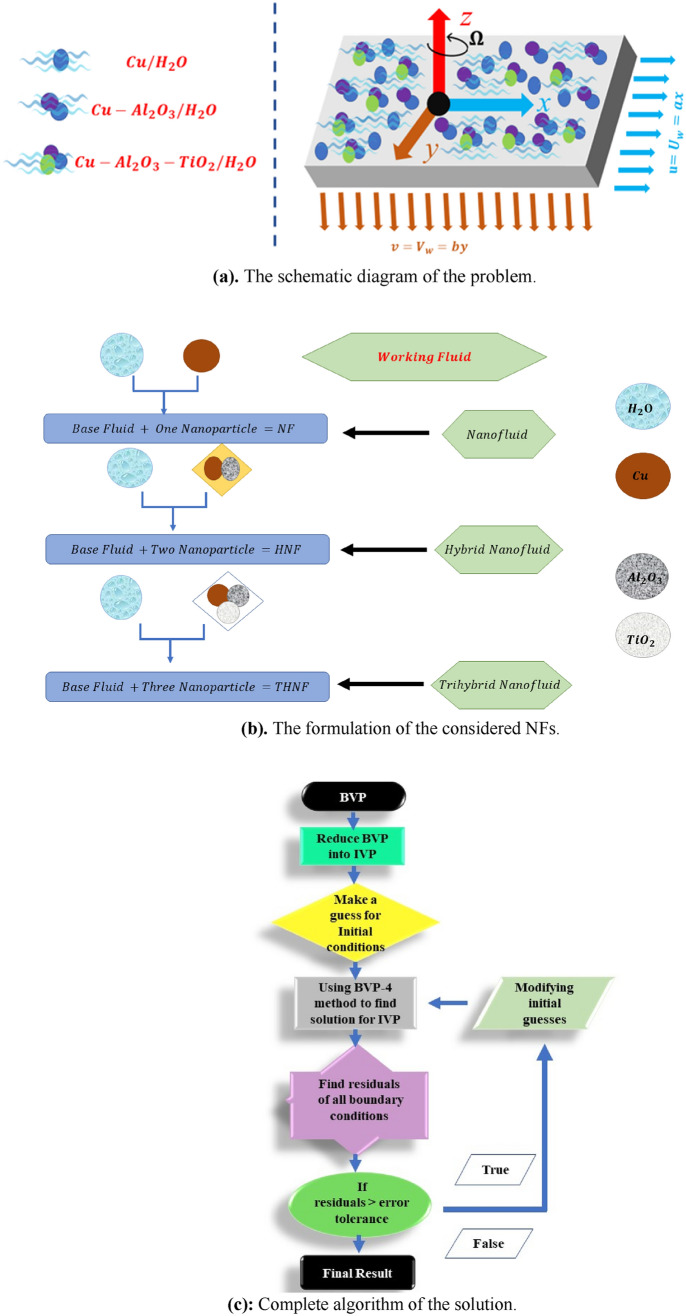
(**a**) The schematic diagram of the problem. (**b**) The formulation of the considered NFs. (**c**) Complete algorithm of the solution.

Let us consider an in-compressible, flow over the stretchable surface with the stretching velocities embedded in a permeable medium. The flow is contained on the surface and the surface is stretchable with surface velocities $u = U_{w} = ax$ and $v = V_{w} = by$ in $x$ and $y$-directions correspondingly. It is assumed that the flow of chemically reactive trihybrid nanofluids is restricted above the plane, in other words, $z > 0$. Based on the situation, the flow assumptions are as follows:

1. The flow is rotational with a constant angular speed ${\Omega }$ and along the *z*-*axis*, as shown in Fig. 1a.
2. We have considered three different types of fluids, namely, NF, HNF, and THNF.
3. Water is used as a working fluid and $Cu/Al_{2} O_{3} / TiO_{2}$^49,50^ as NPs which is presented in Fig. 1b.
4. The surface is inserted in a porous medium and the chemical reaction and thermal radiation effect are considered while modelling.

The thermophysical possessions of NPs and water are defined in Table 1. The flow equations of continuity, momentum, and energy for these assumptions can be presented as:

**Table 1:** Thermophysical^46^ properties of base fluid and nanoparticles.

Physical properties	Density (kg m^−3^)	Specific heat (J kg^−1^ K)	Thermal conductivity (W m^−1^ K)	Thermal expansion (K^−1^)
Water	997	4179	0.614	21 × 10^−5^
Copper (*s*_1_)	8933	385	400	1.67 × 10^−5^
Aluminum oxide (*s*_2_)	3970	765	40	0.85 × 10^−5^
Titanium oxide (*s*_3_)	4250	686	8.96	0.90 × 10^−5^

Continuity equation^51^:

4 $$\frac{\partial u}{{\partial x}} + \frac{\partial v}{{\partial y}} + \frac{\partial w}{{\partial z}} = 0,$$

Momentum equation along the *x*-axis^51^:

5 $$\left( {u\frac{\partial u}{{\partial x}} + v \frac{\partial u}{{\partial y}} + w \frac{\partial u}{{\partial z}} - 2{\Omega }v} \right) = \frac{{\mu_{hnf} }}{{\rho_{hnf} }}\left( {\frac{{\partial^{2} u}}{{\partial x^{2} }} + \frac{{\partial^{2} u}}{{\partial y^{2} }} + \frac{{\partial^{2} u}}{{\partial z^{2} }}} \right) + \frac{{g^{*} \left( {\rho B_{t} } \right)_{hnf} }}{{\rho_{hnf} }}\left( {T - T_{\infty } } \right) - \frac{{\mu_{hnf} }}{{\rho_{hnf} }}\frac{u}{{k_{o} }},$$

Momentum equation along $y$-axis^51^:

6 $$\left( {u\frac{\partial v}{{\partial x}} + v \frac{\partial v}{{\partial y}} + w \frac{\partial v}{{\partial z}} - 2{\Omega }u} \right) = \frac{{\mu_{hnf} }}{{\rho_{hnf} }}\left( {\frac{{\partial^{2} v}}{{\partial x^{2} }} + \frac{{\partial^{2} v}}{{\partial y^{2} }} + \frac{{\partial^{2} v}}{{\partial z^{2} }}} \right) + \frac{{g^{*} \left( {\rho B_{t} } \right)_{hnf} }}{{\rho_{hnf} }}\left( {T - T_{\infty } } \right) - \frac{{\mu_{hnf} }}{{\rho_{hnf} }}\frac{v}{{k_{o} }},$$

Energy equation^51^:

7 $$u\frac{\partial T}{{\partial x}} + v \frac{\partial T}{{\partial y}} + w\frac{\partial T}{{\partial z}} = \alpha_{hnf} \left( { \frac{{\partial^{2} T}}{{\partial x^{2} }} + \frac{{\partial^{2} T}}{{\partial y^{2} }} + \frac{{\partial^{2} T}}{{\partial z^{2} }}} \right) - \frac{1}{{\left( {\rho C_{p} } \right)_{hnf} }}\frac{{\partial q_{r} }}{\partial z}$$

Here $u,v$, and $w$ are velocity components in the $x,y$, and $z$ directions respectively. $T$ is temperature,$k_{0} , q_{r} , g^{*}$ is the porosity, radiation, and gravitational acceleration respectively. $\rho_{hnf} ,\mu_{hnf} , \upalpha _{hnf}$ is the density, viscosity, and thermal diffusivity of a hybrid nanofluid. By applying the Rosseland approximation, the radiative heat flux $q_{r}$ is defined by:

8 $$q_{r} = - \frac{{4\sigma^{*} }}{{3k_{1} }}\frac{{\partial T^{4} }}{\partial z}.$$

Here $\sigma^{*}$ is the Stefan-Boltzmann coefficient and $k_{1}$ is the mean absorption constant. This model considers optically thick radiation. Assuming that there is not much of a temperature differential within the flow, the expression of the term $T^{4}$ by using Taylor series expansion is expanded as follows:

9 $$T^{4} = T^{4}_{\infty } + 4T^{3}_{\infty } \left( {T - T_{\infty } } \right) + 6T^{2}_{\infty } \left( {T - T_{\infty } } \right)^{2} + \cdots$$

Now by neglecting higher order terms beyond the first degree in $\left( {T - T_{\infty } } \right)$, we get.

10 $$T^{4} = 4T^{3}_{\infty } T - 3T^{4}_{\infty } .$$

By using Eqs. (9) and (10), the relation takes the form:

11 $$\frac{{\partial q_{r} }}{\partial z} = - \frac{{16\sigma^{*} T_{\infty }^{3} }}{{3k_{1} }}\frac{{\partial^{2} T}}{{\partial z^{2} }},$$

Therefore, the energy equation takes the form:

12 $$\frac{\partial T}{{\partial x}} + v \frac{\partial T}{{\partial y}} + w\frac{\partial T}{{\partial z}} = \alpha_{hnf} \left( { \frac{{\partial^{2} T}}{{\partial x^{2} }} + \frac{{\partial^{2} T}}{{\partial y^{2} }} + \frac{{\partial^{2} T}}{{\partial z^{2} }}} \right) + \frac{{16\sigma^{*} T_{\infty }^{3} }}{{3k_{1} \left( {\rho C_{p} } \right)_{hnf} }}\frac{{\partial^{2} T}}{{\partial z^{2} }}$$

The corresponding boundary conditions^48^ are:

13 $$\left. {\begin{array}{*{20}r} \hfill {u = U_{w} = ax,\quad v = V_{w} = by,\quad w = 0,\quad T = T_{w} ,} & \hfill {\quad at\;\; z = 0} \\ \hfill {u \to 0,\quad v \to 0,\quad T \to T_{\infty } , } & \hfill {\quad as\;\;z \to \infty } \\ \end{array} } \right\}$$

## Transformation methodology

We define the following transformation^46^:

14 $$\begin{aligned} & u = axp^{\prime}\left( \eta \right), \quad v = ayq^{\prime}\left( \eta \right), \quad w = \sqrt {av_{f} } \left\{ {p\left( \eta \right) + q\left( \eta \right)} \right\}, \\ & r\left( \eta \right)\left( {T_{o} - T_{\infty } } \right) = T - T_{\infty } , \quad \eta = z\sqrt {\frac{a}{{v_{f} }}} . \\ \end{aligned}$$

Here differential is concerned $\eta$. Using this similarity transformation Eq. (14), the Eq. (4) is satisfied. Equations (5), (6), and Eq. (12) take the following transformed form for HNF:

15 $$\begin{aligned} p^{\prime\prime\prime}\left( \eta \right) & = \frac{{\rho_{hnf} }}{{\rho_{f} }} \times \left\{ {p^{\prime}\left( \eta \right)^{2} \left( {p^{\prime}\left( \eta \right) + q^{\prime}\left( \eta \right)} \right) - 2 \lambda \delta q^{\prime}\left( \eta \right) + Z p^{\prime}\left( \eta \right) - \epsilon_{x} \times \theta \times \frac{{\left( {\rho B_{t} } \right)_{hnf} }}{{\left( {\rho B_{t} } \right)_{f} }}} \right\} \\ & \quad \times \left[ {1 - \left( {\phi_{1} + \phi_{2} } \right)} \right]^{5/2} \\ \end{aligned}$$

16 $$\begin{aligned} q^{\prime\prime\prime}\left( \eta \right) & = \frac{{\rho_{hnf} }}{{\rho_{f} }} \times \left\{ {q^{\prime}\left( \eta \right)^{2} \left( {p^{\prime}\left( \eta \right) + q^{\prime}\left( \eta \right)} \right) - 2\frac{\lambda }{\delta } p^{\prime}\left( \eta \right) + Z q^{\prime}\left( \eta \right) - \epsilon_{y} \times \theta \times \frac{{\left( {\rho B_{t} } \right)_{hnf} }}{{\left( {\rho B_{t} } \right)_{f} }}} \right\} \\ & \quad \times \left[ {1 - \left( {\phi_{1} + \phi_{2} } \right)} \right]^{5/2} \\ \end{aligned}$$

17 $$r^{\prime\prime} = - \left[ {\left( {1 + \frac{4}{{3 \times \frac{{k_{hnf} }}{{k_{f} }}}} \times \pi } \right) \times Pr \times \frac{{\left( {\rho C_{p} } \right)_{hnf} }}{{\left( {\rho C_{p} } \right)_{f} }} \times r^{\prime} \times \left( {p\left( \eta \right) + q\left( \eta \right)} \right)} \right]$$

The non-dimensional quantities $\lambda ,\delta , Z,\epsilon , Pr,$ and $\pi$ are defined as and the thermophysical formulation relations are defined in Table 2 for NF, in Table 3 for HNF, and in Table 4 for THNF.

18 $$\begin{aligned} & \lambda = \frac{{\Omega }}{a},\delta = \frac{y}{x}, Z = \frac{{\mu_{hnf} }}{{a\rho_{hnf} k_{o} }},\epsilon_{x} = \frac{{{\text{ Gr}}_{x} }}{{Re_{x}^{2} }},\epsilon_{y} = \frac{{{\text{ Gr}}_{y} }}{{Re_{y}^{2} }} Pr = \frac{{v_{f} }}{{k_{f} }}, \pi = \frac{{4\sigma^{*} T_{\infty }^{3} }}{{k_{1} k_{f} }} \\ & {\mathrm{Here}}\;{\mathrm{Gr}}_{x} = \frac{{g^{*} \left( {\rho B_{t} } \right)_{hnf} }}{{v_{f}^{2} }}\left( {T - T_{\infty } } \right) x^{3} , Re_{x} = \frac{{u_{w} \left( x \right)}}{{v_{f} }} \\ & {\mathrm{and}}\;{\mathrm{Gr}}_{y} = \frac{{g^{*} \left( {\rho B_{t} } \right)_{hnf} }}{{v_{f}^{2} }}\left( {T - T_{\infty } } \right) y^{3} , Re_{y} = \frac{{v_{w} \left( y \right)}}{{v_{f} }} \\ \end{aligned}$$

**Table 2:** Thermophysical^46^ formulation for nanofluid (NF).

Properties	Nanofluid
Density	$\rho_{nf} = \left( {1 - (\phi_{1} } \right)) \times \rho_{f} + \phi_{1} \times \rho_{s1} ,$
Dynamic viscosity	$\mu_{nf} = \frac{{\mu_{f} }}{{\left[ {1 - \left( {\phi_{1} } \right)} \right]^{5/2} }},$
Heat capacity	$\left( {\rho C_{p} } \right)_{nf} = \left[ {1 - \left( {\phi_{1} } \right)} \right] \times \left( {\rho c_{p} } \right)_{f} + \phi_{1} \times \left( {\rho c_{p} } \right)_{s1} ,$
Thermal expansion	$\left( {\rho B_{t} } \right)_{nf} = \left( {1 - (\phi_{1} } \right)) \times \rho B_{tf} + \phi_{1} \times \rho B_{ts1} ,$
Thermal conductivity	$\frac{{k_{nf} }}{{k_{f} }} = \frac{{k_{s1} + 2k_{f} - 2\phi_{1} \left( {k_{f} - k_{s1} } \right)}}{{k_{s1} + 2k_{f} + \phi_{1} \times \left( {k_{f} - k_{s1} } \right)}},$

**Table 3:** Thermophysical^46^ formulation for hybrid nanofluid.

Properties	Hybrid nanofluid
Density	$\rho_{hnf} = \left[ {1 - (\phi_{2} )} \right] \times \left[ {1 - (\phi_{1} ) \times \rho_{f} + \phi_{1} \times \rho_{s1} } \right] + \phi_{2} \times \rho_{s2} ,$
Dynamic viscosity	$\mu_{hnf} = \frac{{\mu_{f} }}{{\left[ {1 - \left( {\phi_{1} + \phi_{2} } \right)} \right]^{5/2} }},$
Heat capacity	$\left( {\rho C_{p} } \right)_{hnf} = \left[ {1 - (\phi_{2} )} \right] \times \left[ {1 - (\phi_{1} ) \times \left( {\rho C_{p} } \right)_{f} + \phi_{1} \times \left( {\rho C_{p} } \right)_{s1} } \right] + \phi_{2} ,$
Thermal expansion	$\left( {\rho B_{t} } \right)_{hnf} = \left[ {1 - (\phi_{2} )} \right] \times \left[ {1 - (\phi_{1} ) \times \left( {\rho B_{t} } \right)_{f} + \phi_{1} \times \left( {\rho B_{t} } \right)_{s1} } \right] + \phi_{2} ,$
Thermal conductivity	$\frac{{k_{hnf} }}{{k_{nf} }} = \frac{{k_{s2} + 2 \times k_{nf} - 2 \times \phi_{2} \times \left( {k_{nf} - k_{s2} } \right)}}{{k_{s2} + 2 \times k_{nf} + \phi_{2} \times \left( {k_{nf} - k_{s2} } \right)}},$ Here $\frac{{k_{nf} }}{{k_{f} }} = \frac{{k_{s1} + 2 \times k_{f} - 2 \times \phi_{1} \times \left( {k_{f} - k_{s1} } \right)}}{{k_{s1} + 2 \times k_{f} + \phi_{1} \times \left( {k_{f} - k_{s1} } \right)}}$

**Table 4:** Thermophysical^52^ formulation for trihybrid nanofluid.

Properties	Tri-Hybrid nanofluid
Density	$$\begin{aligned} \rho_{thnf} & = \left[ {1 - (\phi_{1} )} \right] \times \left[ {\left[ {1 - (\phi_{2} )} \right] \times \left\{ {\left[ {1 - (\phi_{3} )} \right] + \phi_{3} \times \rho_{s3} } \right\} + \phi_{2} \times \rho_{s2} } \right] \\ & \quad + \phi_{1} \times \rho_{s1} , \\ \end{aligned}$$
Dynamic viscosity	$\mu_{hnf} = \frac{{\mu_{f} }}{{\left[ {1 - \left( {\phi_{1} + \phi_{2} + \phi_{3} } \right)} \right]^{5/2} }},$
Heat capacity	$$\begin{aligned} \left( {\rho C_{p} } \right)_{thnf} & = \left[ {1 - (\phi_{1} )} \right] \\ & \quad \times \left[ {\left[ {1 - (\phi_{2} )} \right] \times \left\{ {\left[ {1 - (\phi_{3} )} \right] + \phi_{3} \times \left( {\rho C_{p} } \right)_{s3} } \right\} + \phi_{2} \times \left( {\rho C_{p} } \right)_{s2} } \right] \\ & \quad + \phi_{1} \times \left( {\rho C_{p} } \right)_{s1} , \\ \end{aligned}$$
Thermal expansion	$$\begin{aligned} \left( {\rho C_{p} } \right)_{thnf} & = \left[ {1 - (\phi_{1} )} \right] \\ & \quad \times \left[ {\left[ {1 - (\phi_{2} )} \right] \times \left\{ {\left[ {1 - (\phi_{3} )} \right] + \phi_{3} \times \left( {\rho B_{t} } \right)_{s3} } \right\} + \phi_{2} \times \left( {\rho B_{t} } \right)_{s2} } \right] \\ & \quad + \phi_{1} \times \left( {\rho B_{t} } \right)_{s1} , \\ \end{aligned}$$
Thermal conductivity	$\frac{{k_{thnf} }}{{k_{hnf} }} = \frac{{k_{s1} + 2 \times k_{hnf} - 2 \times \phi_{1} \times \left( {k_{hnf} - k_{s1} } \right)}}{{k_{s1} + 2 \times k_{hnf} + \phi_{1} \times \left( {k_{hnf} - k_{s1} } \right)}},$ Here $\frac{{k_{hnf} }}{{k_{nf} }} = \frac{{k_{s2} + 2 \times k_{nf} - 2 \times \phi_{2} \times \left( {k_{nf} - k_{s2} } \right)}}{{k_{s2} + 2 \times k_{nf} + \phi_{2} \times \left( {k_{nf} - k_{s2} } \right)}},$ and $\frac{{k_{nf} }}{{k_{f} }} = \frac{{k_{s3} + 2 \times k_{f} - 2 \times \phi_{3} \times \left( {k_{f} - k_{s3} } \right)}}{{k_{s3} + 2 \times k_{f} + \phi_{3} \times \left( {k_{f} - k_{s3} } \right)}}$

The modified boundary conditions are as follows:

19 $$\left. {\begin{array}{*{20}r} \hfill {p = 0, \quad p^{\prime} = 1,\quad q = 0,\quad q^{\prime} = \gamma ,\quad r = 1,} & \hfill {\quad at\;\;\eta = 0} \\ \hfill {p^{\prime} \to 0, \quad q^{\prime} \to 0, \quad r \to 0,} & \hfill {\quad as\;\;\eta \to \infty } \\ \end{array} } \right\}$$

Here $\gamma = \frac{b}{a}$ is the dimensionless stretching ratio parameter.

## Quantities of engineering interest

The most significant physical quantities of importance from an engineering perspective are the skin friction coefficients $Cf_{x}$, $Cf_{y}$, and Nusselt number $Nu$, which are defined as follows:

20 $$Cf_{x} = \frac{{\tau_{zx} }}{{\rho_{f} u_{w}^{2} }}, \quad Cf_{y} = \frac{{\tau_{zy} }}{{\rho_{f} v_{w}^{2} }}$$

Here $\tau_{zx}$ and $\tau_{zy}$ denote the shear stress along the stretched wall along the *x*-*axis* and *y*-*axis* and are defined as

21 $$\tau_{zx} = \mu_{hnf} \left( {\frac{\partial u}{{\partial z}} + \frac{\partial w}{{\partial x}}} \right)_{z = 0} ,\quad \tau_{zy} = \mu_{hnf} \left( {\frac{\partial v}{{\partial z}} + \frac{\partial w}{{\partial y}}} \right)_{z = 0}$$

The dimensionless form of Eq. (18) is:

22 $$\begin{aligned} \left( {Re_{x} } \right)^{\frac{1}{2}} Cf_{x} & = \frac{1}{{\left[ {1 - \left( {\phi_{1} + \phi_{2} } \right)} \right]^{\frac{5}{2}} }} \times p^{\prime\prime}\left( 0 \right), \\ \left( {Re_{x} } \right)^{\frac{1}{2}} Cf_{y} & = \frac{1}{{\left[ {1 - \left( {\phi_{1} + \phi_{2} } \right)} \right]^{\frac{5}{2}} }} \times q^{\prime\prime}\left( 0 \right), \\ \end{aligned}$$

By using the temperature field to define the thermal transmission rate as a Nusselt number:

23 $$Nu_{x} = \frac{{xq_{w} }}{{k_{f} \left( {T_{w} - T_{\infty } } \right)}},\quad q_{w} = - k_{hnf} \left( {\frac{\partial T}{{\partial z}}} \right)_{z = 0} + (q_{r} )_{w} ,$$

Or

24 $$Nu_{x} = - \left( {\frac{{k_{hnf} }}{{k_{f} }} + \frac{4}{3}\pi } \right)r^{\prime}\left( 0 \right),$$

## Numerical scheme and validation

The numerical outcomes for the present study are obtained through the bvp-4c at MATLAB. The complete algorithm in graphical form is presented in Fig. 1c. The Eqs. (15)–(17) which are highly non-linear are changed to first-order ODEs by using a new set of variables. Subsequently, the initial guesses are chosen which satisfy the boundary conditions. A convergence criterion of $10^{ - 6}$ is set for the acquired solution.

25 $$\begin{aligned} & y_{3}{\prime} = p^{\prime\prime\prime},\quad y_{3} = p^{\prime\prime},\quad y_{2} = p^{\prime},\quad y_{1} = p, \\ & y_{6}{\prime} = q^{\prime\prime\prime},\quad y_{6} = q^{\prime\prime},\quad y_{5} = q^{\prime},\quad y_{4} = q, \\ & y_{8}{\prime} = r^{\prime\prime},\quad y_{8} = r^{\prime},\quad y_{7} = r, \\ \end{aligned}$$

A new set of variables are defined as follows in MATLAB to obtain the numerical solution.

26 $$y_{1}{\prime} = y_{2}$$

27 $$y_{2}{\prime} = y_{3}$$

28 $$\begin{aligned} y_{3}{\prime} & = \frac{{\rho_{hnf} }}{{\rho_{f} }} \times \left\{ {\left[ {y_{2}^{2} \times \left( {y_{1} + y_{4} } \right)} \right] - \left[ {2 \times \lambda \times \delta \times y_{5} } \right] + \left[ {Z \times y_{2} } \right] - \left[ {\epsilon_{x} \times y_{7} \times \frac{{\left( {\rho B_{t} } \right)_{hnf} }}{{\left( {\rho B_{t} } \right)_{f} }}} \right]} \right\} \\ & \quad \times \left[ {1 - \left( {\phi_{1} + \phi_{2} } \right)} \right]^{5/2} \\ \end{aligned}$$

29 $$y_{4}{\prime} = y_{5}$$

30 $$y_{5}{\prime} = y_{6}$$

31 $$\begin{aligned} y_{6}{\prime} & = \frac{{\rho_{hnf} }}{{\rho_{f} }} \times \left\{ {\left[ {y_{5}^{2} \times \left( {y_{4} + y_{1} } \right)} \right] - \left[ {2 \times \lambda \times \frac{1}{\delta } \times y_{2} } \right] + \left[ {Z \times y_{5} } \right] - \left[ {\epsilon_{y} \times y_{7} \times \frac{{\left( {\rho B_{t} } \right)_{hnf} }}{{\left( {\rho B_{t} } \right)_{f} }}} \right]} \right\} \\ & \quad \times \left[ {1 - \left( {\phi_{1} + \phi_{2} } \right)} \right]^{5/2} \\ \end{aligned}$$

32 $$y_{7}{\prime} = y_{8}$$

33 $$y_{8}{\prime} = - \left[ {\left( {1 + \frac{4}{{3 \times \frac{{k_{hnf} }}{{k_{f} }}}} \times \pi } \right) \times Pr \times \frac{{\left( {\rho C_{p} } \right)_{hnf} }}{{\left( {\rho C_{p} } \right)_{f} }} \times y_{8} \times \left( {y_{1} + y_{4} } \right)} \right]$$

The transformed boundary conditions changed into the subsequent form:

34 $$\left. {\begin{array}{*{20}r} \hfill {y_{1} = 0,\quad y_{2} = 1,\quad y_{4} = 0,\quad y_{5} = \gamma ,\quad y_{7} = 1,} & \hfill {\quad at\;\;\eta = 0} \\ \hfill {y_{2} \to 0,\quad y_{5} \to 0,\quad y_{7} \to 0,} & \hfill {\quad as\;\;\eta \to \infty } \\ \end{array} } \right\}$$

The skin friction and Nusselt number changed into the following form:

35 $$\begin{aligned} \left( {Re_{x} } \right)^{\frac{1}{2}} Cf_{x} & = \frac{1}{{\left[ {1 - \left( {\phi_{1} + \phi_{2} } \right)} \right]^{\frac{5}{2}} }} \times y_{3} \left( 0 \right), \\ \left( {Re_{x} } \right)^{\frac{1}{2}} Cf_{y} & = \frac{1}{{\left[ {1 - \left( {\phi_{1} + \phi_{2} } \right)} \right]^{5/2} }} \times y_{6} \left( 0 \right), \\ \end{aligned}$$

36 $$Nu_{x} = - \left( {\frac{{k_{hnf} }}{{k_{f} }} + \frac{4}{3} \times \pi } \right) \times y_{8} \left( 0 \right).$$

The attained results present the influence on different profiles. The results are assessed with the literature in Table 5.

**Table 5:** The assessment of the present numerical outcomes with literature.

$\lambda$	Wang^53^		Present outcomes and^54^		Nazar et al.^55^	
	$p^{\prime\prime}\left( 0 \right)$	$q^{\prime\prime}\left( 0 \right)$	$p^{\prime\prime}\left( 0 \right)$		$p^{\prime\prime}\left( 0 \right)$	$q^{\prime\prime}\left( 0 \right)$
0.1	− 1.0	$q^{\prime\prime}\left( 0 \right)$	− 1.0	0.0	− 1.0	0.0
0.5	− 1.13	0.0	− 1.145	− 0.569	− 1.13	− 0.51
1.0	− 1.32	− 0.51	− 1.334	− 0.888	− 1.32	− 0.83
2.0	− 1.65	− 0.83	− 1.661	− 1.3285	− 1.65	− 1.28

## Results and discussion

The formulation of the problem presented in the preceding section is for HNF. The outputs of the present problem for NF, HNF, and THNF are obtained separately for each nanofluid i.e., copper–water NF, copper/aluminum oxide–water HNF, and copper/aluminum oxide/titanium oxide–water THNF. The effects of different parameters by employing the boundary value problem technique at MATLAB are described in this section.

### Velocity profile

**Figure 2:**
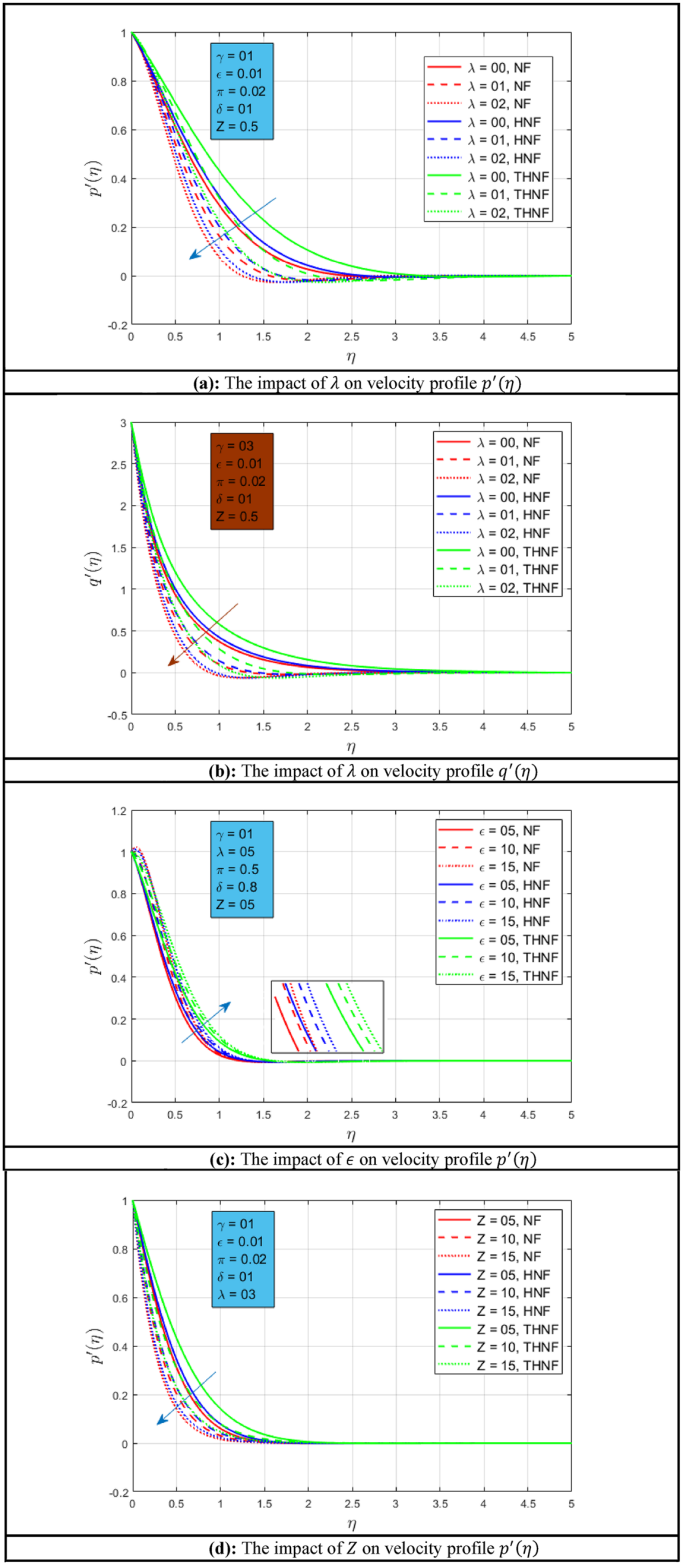

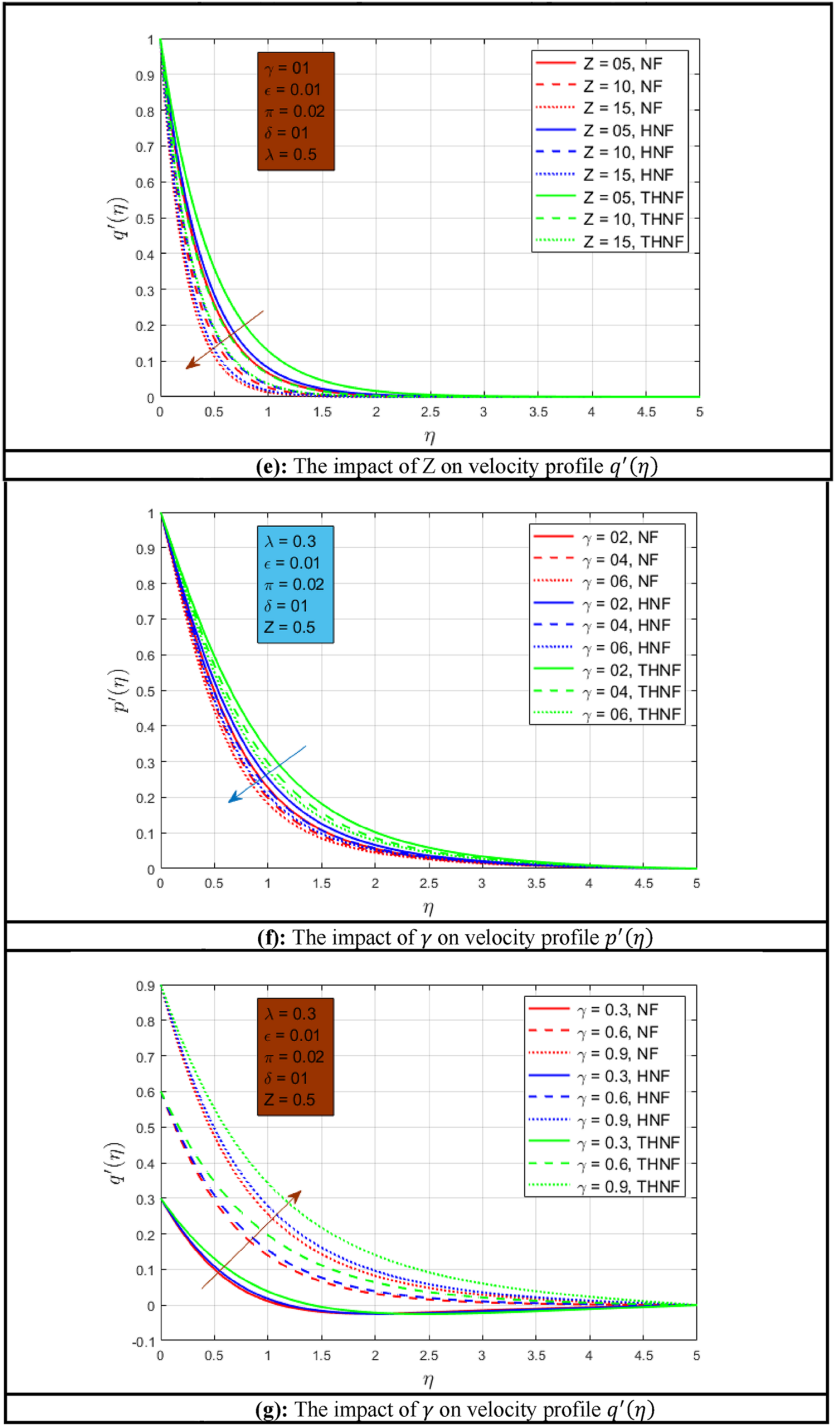
(**a**) The impact of $\lambda$ on velocity profile $p^{\prime}\left( \eta \right)$. (**b**) The impact of $\lambda$ on velocity profile $q^{\prime}\left( \eta \right)$. (**c**) The impact of $\epsilon$ on velocity profile $p^{\prime}\left( \eta \right)$. (**d**) The impact of $Z$ on velocity profile $p^{\prime}\left( \eta \right)$. (**e**) The impact of Z on velocity profile $q^{\prime}\left( \eta \right)$. (**f**) The impact of $\gamma$ on velocity profile $p^{\prime}\left( \eta \right)$. (**g**) The impact of $\gamma$ on velocity profile $q^{\prime}\left( \eta \right)$.

The impact of rotation parameters on velocity profiles $p^{\prime}\left( \eta \right)$ and $q^{\prime}\left( \eta \right)$ is presented in Fig. 2a,b for NF, HNF, and THNF where water is the host fluid. Figure 2a illustrates the effect of rotational motion on the primary velocity profile $p^{\prime}\left( \eta \right)$. It is examined that when the rotational motion is increased the velocity profile $p{\prime} \left( \eta \right)$ decline for NF, HNF, and THNF. Observations indicate form that $p^{\prime}\left( \eta \right)$ has an inverse relationship with $\lambda$. In addition, the momentum boundary layer has considerably extended under rotation influence in the case of THNF as associated with NF and HNF. The reason behind this phenomenon is that when three nanoparticles are dispersed in base liquid the volumetric concentration increases as compared to NF and HNF. When the influence of rotation on velocity profiles is compared with^46^, it is observed that a wider momentum boundary layer is noted. Figure 2b depicts the influence of rotation on secondary velocity $q^{\prime}\left( \eta \right)$. The velocity in the secondary direction $q^{\prime}\left( \eta \right)$ has declined for all the fluid types under consideration. The significant decline in velocities profiles is the direct relation of $\lambda$ with the rotational angular velocity ${\Omega }$ of flow. The change of mixed convection constraint $\epsilon$ on velocity profile $p^{\prime}\left( \eta \right)$ is shown in Fig. 2c for all types of fluids considered in this comparative study. The velocity profile $p^{\prime}\left( \eta \right)$ has a direct relation with mixed convection constraint as shown in this Fig. When mixed convection occurs, the buoyancy on free convection becomes significant. That’s why if the mixed convection constraint increases the buoyancy will increase and as a result, the velocity profiles will increase. The lower momentum boundary layer is noted for NF and higher for THNF. When the influence of the mixed convection parameter is compared with^46^, it is observed that a thinner momentum boundary layer is noted. Figure 2d,e show the effect of porosity constraint on velocity profile $p^{\prime}\left( \eta \right)$ and $q^{\prime}\left( \eta \right)$ respectively. Here also a similar trend is noted for NF, HNF, and THNF for a rise in porous medium parameters. The velocity profiles $p^{\prime}\left( \eta \right)$ and $q^{\prime}\left( \eta \right)$ decay as the permeability of the medium increases. This is due to the permeability of the stretchable surface. When the influence of the porosity parameter is compared with^46^, it is observed that a similar momentum boundary layer is noted. The effect of stretching ratio constraint $\gamma$ on profiles $p^{\prime}\left( \eta \right)$ and $q^{\prime}\left( \eta \right)$ is presented in Fig. 2f,g. It can be observed that when the stretching rate in the y-direction raises the velocity profile $p^{\prime}\left( \eta \right)$ declines and when the stretching rate increases along the x-axis, the velocity profile $q^{\prime}\left( \eta \right)$ improves and the wide momentum boundary layer is observed for THNF.

### Temperature profile

**Figure 3:**
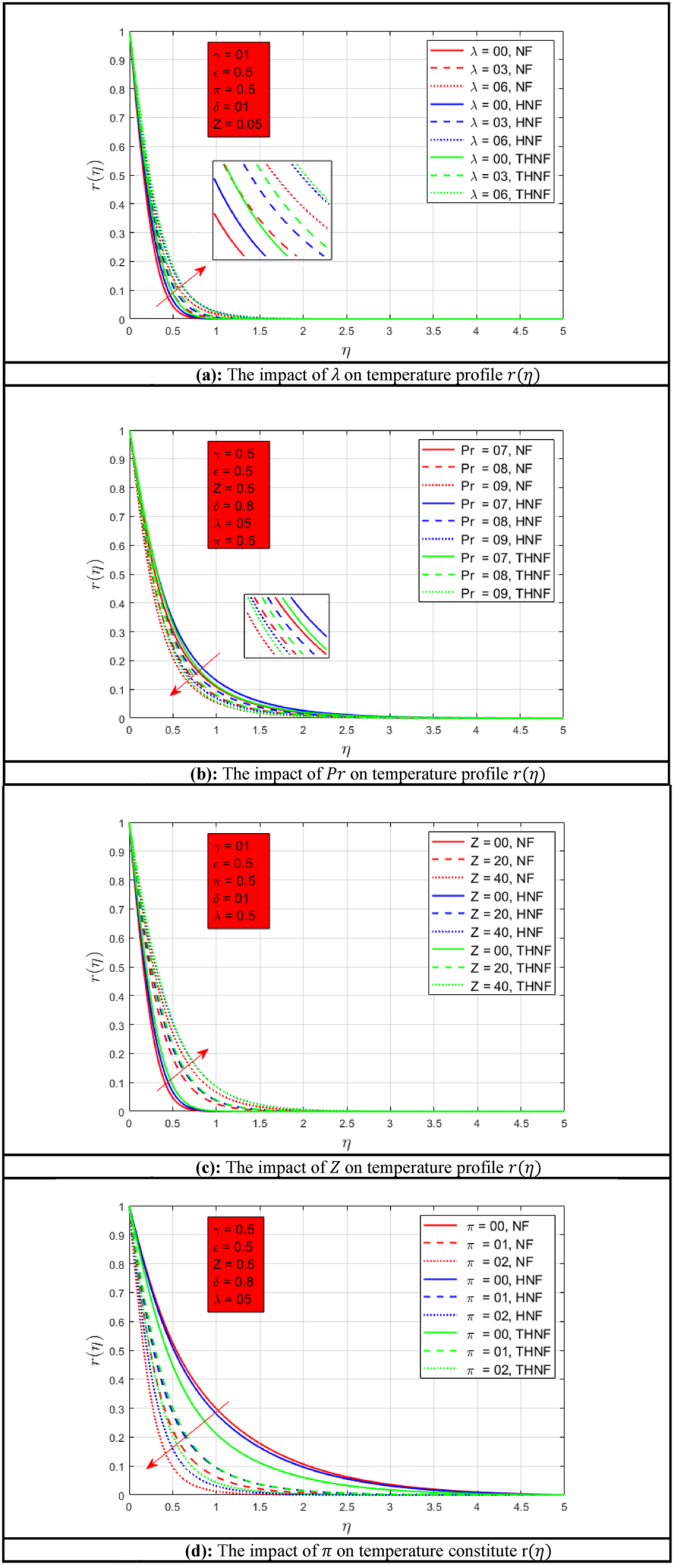

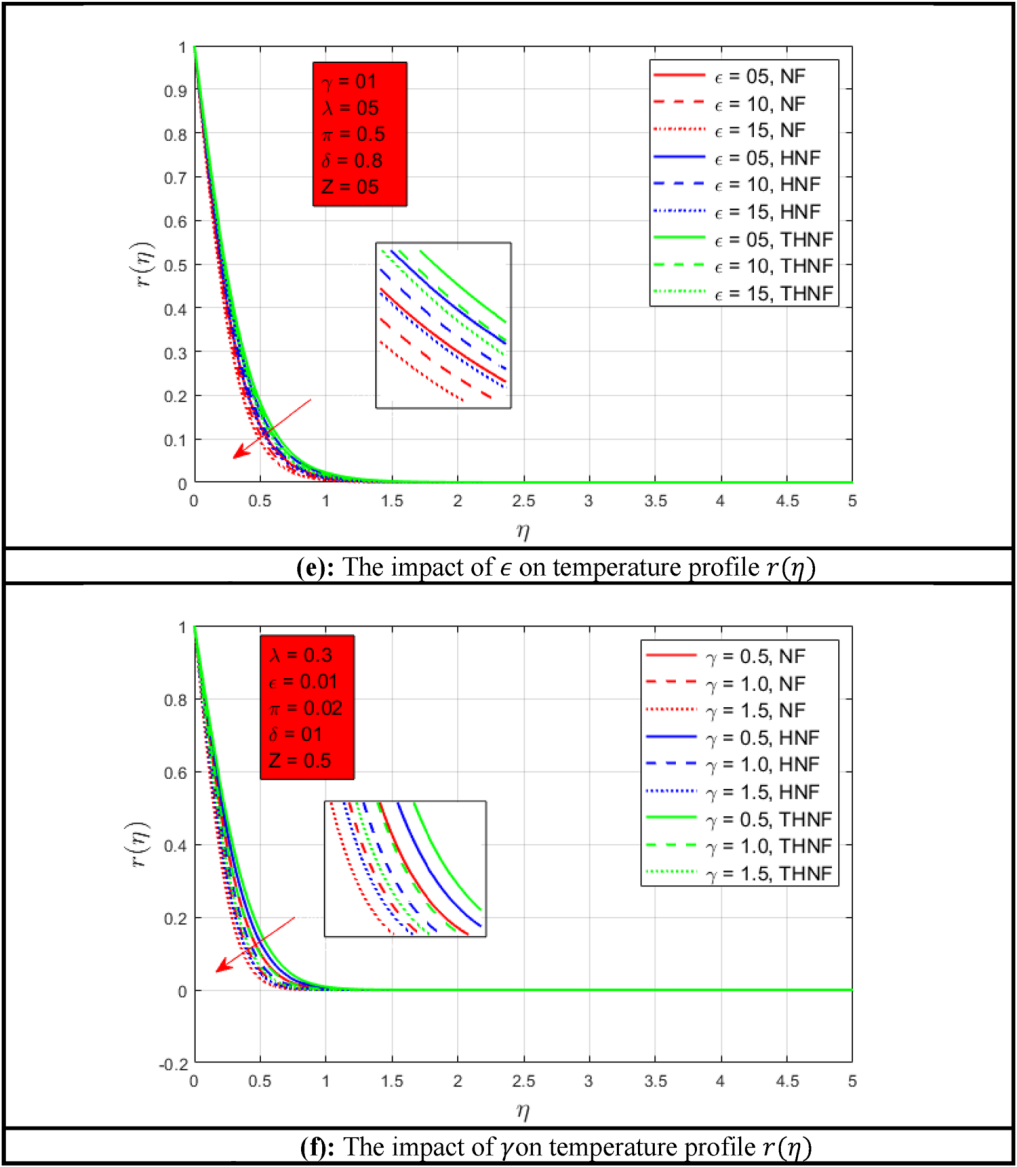
(**a**) The impact of $\lambda$ on temperature profile $r\left( \eta \right)$. (**b**) The impact of $Pr$ on temperature profile $r\left( \eta \right)$. (**c**) The impact of $Z$ on temperature profile $r\left( \eta \right)$. (**d**) The impact of $\pi$ on temperature constitute r$\left( \eta \right)$. (**e**) The impact of $\epsilon$ on temperature profile $r\left( \eta \right)$. (**f**) The impact of $\gamma$ on temperature profile $r\left( \eta \right)$.

The following Figures show the influence of different parameters on temperature profile $r\left( \eta \right)$. Figure 3a shows the influence of rotation parameter $\lambda$ on temperature profile $r\left( \eta \right)$. As the rotation of the NFs enhances the heat transfer rate of NF, HNF, and THNF increases. A higher rate of heat transmission is observed for THNF due to the combination of three nanoparticles. Comparatively, a thinner energy profile is noted when compared with^46^. The effect of Prandtl number $Pr$ on temperature profile $r\left( \eta \right)$ is presented in Fig. 3b. The Prandtl number and temperature profile $r\left( \eta \right)$ have inverse relations as depicted in Figure. Temperature profile $r\left( \eta \right)$ decays as the Prandtl number increases for NF, HNF, and THNF nanofluid. However, a consistent thermal boundary layer is examined for THNF. The Prandtl number has a greater influence on energy profile as compared to^46^. Permeability effects on temperature profile $r\left( \eta \right)$ are shown in Fig. 3c. When porosity $Z$ of the stretchable surface improves, the heat transfer of all types of NFs increases because the fluid flow rate improves. Porosity has a huge difference between the present study and^46^. The change of radiation parameter $\pi$ is presented in Fig. 3d. It can be observed that when the radiation effect is zero, the thermal boundary layers a closer to each other for all types of nanofluids. Suddenly, when the radiation parameter $\pi$ increases the temperature boundary layers get closer due to resistance which does not allow the fluid to flow easily. As an outcome, the temperature profile $r\left( \eta \right)$ reduces when the radiation parameter $\pi$ increases. Comparatively^46^ have a wider energy profile as associated with the present study. The impact of mixed convection constraint $\epsilon$ and stretching rate parameter $\gamma$ is described in Fig. 3e,f respectively. As the mixed convection constraint $\epsilon$ increases the temperature profile $r\left( \eta \right)$ decreases accordingly. The highest rate of heat transfer is observed for THNF. The effect of stretching surface parameter $\gamma$ for temperature profile $r\left( \eta \right)$ is represented in Fig. 3f. The higher stretching rate along the *y*-*axis*, *x*-*axis*, and the same stretching rate for NF, HNF, and THNF are presented. The consistent and higher temperature profile $r\left( \eta \right)$ is observed for THNF as compared to other both NFs.

### Skin frictions and Nusselt number

**Figure 4:**
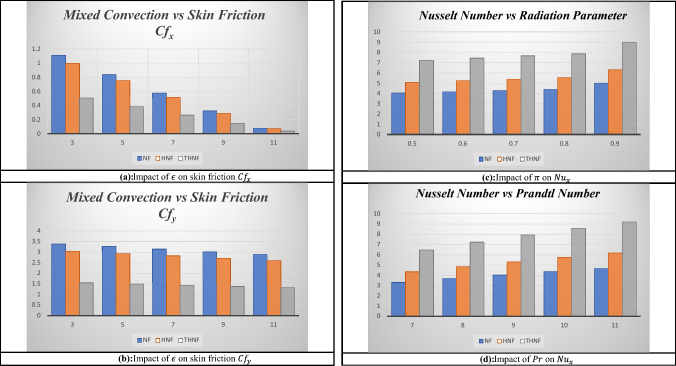
(**a**) Impact of $\epsilon$ on skin friction $Cf_{x}$. (**b**) Impact of $\epsilon$ on skin friction $Cf_{y}$. (**c**) Impact of $\pi$ on $Nu_{x}$. (**d**) Impact of $Pr$ on $Nu_{x}$.

In this section, the solutions for skin frictions $Cf_{x}$, $Cf_{y}$, and Nusselt number $Nu_{x}$ are presented. The influence of mixed convection $\epsilon$ on skin friction along the *x*-*axis* and *y*-*axis* is presented in Fig. 4a,b. These figures show that the skin friction gradually decreases as the mixed convection parameter increases for NF, HNF, and THNF. Higher skin friction along the $y - axis$ is observed as compared to the $x - axis$. Minimum resistance is noted for THNF. The influence of radiation constraint $\pi$ and Prandtl number $Pr$ is presented in Fig. 4c,d. A rise in radiation parameters increases the Nusselt number for all types of NFs. A higher change is observed for THNF in Fig. 4c. Figure 4d shows the influence of the Prandtl number on the Nusselt number for NF, HNF, and THNF. The highest Nusselt number for THNF is noted when the Prandtl number increases as compared to NF and HNF. Table 6 shows the numerical results of skin friction along the *x*-*axis* and the *y*-*axis* and Table 7 shows the numerical outcomes of the Nusselt number for NF, HNF, and THNF by changing the values of different constraints. In the absence of rotation, a maximum heat transfer rate and reduced skin friction are noted. Porosity has a direct relation with heat transfer and skin friction constants. Minimum skin friction and maximum Nusselt number are observed when the surface stretching rate along the *y*-*axis* is high as compared to the *x*-*axis*. As the mixed convection constraint increases the skin friction along both axis and the heat transfer rate increases. The thermal radiation and Prandtl number have the same relation with skin friction and the Nusselt number constant. They both increase by raising the values of thermal radiation and Prandtl number.

**Table 6:** Numerical results of different parameters for skin frictions along *x*-*axis* $Cf_{x}$ and *y*-*axis* $Cf_{y}$ respectively.

$\lambda$	Z	$\gamma$	$\epsilon$	$\pi$	$Pr$	$Cf_{x}$ for NF	$Cf_{y}$ for NF	$Cf_{x}$ for HNF	$Cf_{y} for$ HNF	$Cf_{x}$ for THNF	$Cf_{y}$ for THNF
0.0	0.5	0.8	0.3	0.5	6.2	− 1.6519	− 0.70939	− 1.3778	− 0.596258	− 0.575551	− 0.253026
1.0						− 1.07303	− 1.97035	− 0.898143	− 1.64223	− 0.378037	− 0.684988
2.0						− 0.94837	− 2.92488	− 0.795099	− 2.43386	− 0.335846	− 1.0117
0.7	0.5	0.8	0.3	0.5	6.2	− 1.1588	− 1.61871	− 0.969137	− 1.35058	− 0.407187	− 0.564586
	0.8					− 1.31191	− 1.69061	− 1.09617	− 1.41021	− 0.459695	− 0.589201
	1.0					− 1.40886	− 1.7388	− 1.17662	− 1.45018	− 0.492948	− 0.605703
0.7	0.5	0.9	0.3	0.5	6.2	− 1.10348	− 1.75272	− 0.922987	− 1.46165	− 0.387843	− 0.610427
		1.0				− 1.04933	− 1.89452	− 0.877833	− 1.57915	− 0.368984	− 0.658858
		1.1				− 0.99663	− 2.04558	− 0.833897	− 1.70435	− 0.350649	− 0.710478
0.7	0.5	0.8	0.0	0.5	6.2	− 1.23184	− 1.68788	− 1.02257	− 1.40115	− 0.423177	− 0.579995
			0.3			− 1.15882	− 1.61873	− 0.96915	− 1.35063	− 0.407145	− 0.564684
			0.6			− 1.0864	− 1.55002	− 0.91613	− 1.30039	− 0.391188	− 0.549428
0.7	0.5	0.8	0.3	0.8	6.2	− 1.16542	− 1.62446	− 0.972939	− 1.3539	− 0.407938	− 0.565385
				1.0		− 1.16892	− 1.62756	− 0.975049	− 1.35575	− 0.408404	− 0.565802
				1.2		− 1.17191	− 1.63023	− 0.976906	− 1.35739	− 0.408829	− 0.56618
0.7	0.5	0.8	0.3	0.5	6.2	− 1.15882	− 1.61873	− 0.96915	− 1.35063	− 0.407145	− 0.564684
					7.2	− 1.16333	− 1.62263	− 0.972452	− 1.35348	− 0.408138	− 0.565564
					8.2	− 1.16746	− 1.62626	− 0.97547	− 1.35612	− 0.40904	− 0.56638

**Table 7:** Numerical outcomes of different parameters for Nusselt number $Nu_{x}$.

$\lambda$	Z	$\gamma$	$\epsilon$	$\pi$	$pr$	$Nu_{x}$ for NF	$Nu_{x}$ for HNF	$Nu_{x}$ for THNF
0.0	0.5	0.8	0.3	0.5	6.2	5.65765	6.83338	9.3672
1.0						5.49454	6.63341	9.13085
2.0						5.27139	6.35964	8.81215
0.7	0.5	0.8	0.3	0.5	6.2	5.56119	6.71513	9.22685
	0.8					5.52006	6.66491	9.16575
	1.0					5.49329	6.6322	9.12599
0.7	0.5	0.9	0.3	0.5	6.2	5.74435	6.9371	9.5261
		1.0				5.91972	7.14956	9.81305
		1.1				6.08786	7.35321	10.0886
0.7	0.5	0.8	0.0	0.5	6.2	5.54197	6.69438	9.20895
			0.3			5.56118	6.7151	9.22679
			0.6			5.58889	6.73545	9.24443
0.7	0.5	0.8	0.3	0.8	6.2	9.37348	10.1468	12.3225
				1.0		9.96987	10.6638	12.7641
				1.2		10.5351	11.1587	13.1919
0.7	0.5	0.8	0.3	0.5	6.2	5.56118	6.7151	9.22679
					7.2	5.98464	7.2274	9.92313
					8.2	6.4251	7.7602	10.64739

## Conclusions

In this investigation, the authors studied three-dimensional, rotating, incompressible, NF, HNF, and THNF above permeable stretchable surfaces for the heat transmission rate. The major implications are as follows:

1. The velocity profile $p^{\prime}\left( \eta \right)$ declines with a rising in stretching ratio, rotation, and porosity parameters.
2. The velocity profile $q^{\prime}\left( \eta \right)$ increases with a rise in the stretching ratio parameter while it decays with a rise in porosity and rotation.
3. The temperature profile $r\left( \eta \right)$ declines by an increase in stretching ratio, radiation, Prandtl number, and mixed convection parameter while it rises by increasing the rotation and porous medium parameter.
4. The skin friction decays when mixed convection constraint increases and higher skin friction along the *y*-*axis* is noted when compared to the *x*-*axis* skin friction.
5. The Nusselt number increases by rising the radiation constraint and the Prandtl number increases. Nusselt numbers have increased by 50% in the case of tri-hybrid nanoparticles when compared with hybrid and mono particles.
6. Reduced skin friction and higher heat transfer rate are noted for triple nanoparticle nanofluid.

*Future recommendations* In the future, this study can be extended to discuss heat and mass transfer by selecting the mixed combination of solid NPs and oxide NPs by using different base fluids like ethylene glycol, engine oil, etc.

## Data Availability

All data generated or analyzed during this study are included in this published article.

## List of symbols

$${\Omega }$$: Angular speed
$$C_{p}$$: Specific heat
$\phi_{1} ,\phi_{2} ,\phi_{3}$: Volume fraction of nanoparticles
$$\eta$$: Similarity variable
$$T$$: Temperature
$$Cf_{x} , Cf_{y}$$: Skin frictions
$a,b$: Stretching rate along x and y axis
$$Pr$$: Prandtl number
$$r$$: Temperature profile
$$q_{w}$$: Heat flux
*bvp-4c*: Boundary value problem
*R*: Reynolds number
$$g^{*}$$: Gravitational acceleration
$\gamma$: Stretching ratio parameter
$$k$$: Thermal conductivity
$Nu_{x}$: Nusselt number coefficient
$$NF$$: Nanofluid
$u,v$*, w*: Velocity components in $x,y,z$ direction
*Z*: Porosity parameter
$$p^{\prime}, q^{\prime}$$: Dimensionless velocity
$${\mathrm{HNF}}$$: Hybrid nanofluid
$${\mathrm{THNF}}$$: Trihybrid nanofluid
$$v_{f}$$: Kinematic viscosity
$$\mu_{f}$$: Dynamic viscosity
ODE: Ordinary differential equation
3D: Three dimensional

## Greek symbols

$$\alpha$$: Temperature diffusivity
$${\uplambda }$$: Rotational velocity
$$\rho$$: Density
$\pi$: Thermal radiation parameter
$$\tau_{w}$$: Shear stress
$\epsilon$: Mixed convection parameter
