# Correction: Comparative dynamics of mixed convection heat transfer under thermal radiation effect with porous medium flow over dual stretched surface

**DOI:** 10.1038/s41598-026-61028-1

**Published:** 2026-07-16

**Authors:** Mohammad Mahtab Alam, Mubashar Arshad, Fahad M. Alharbi, Ali Hassan, Qusain Haider, Laila A. Al-Essa, Sayed M. Eldin, Abdulkafi Mohammed Saeed, Ahmed M. Galal

**Affiliations:** 1 Department of Basic Medical Sciences, College of Applied Medical Science, King Khalid University, 61421 Abha, Saudi Arabia; 2 Department of Mathematics, University of Gujrat, Gujrat, 50700 Pakistan; 3 Department of Mathematics, Al-Qunfudah University College, Umm Al-Qura University, Mecca, Saudi Arabia; 4 Department of Mathematical Sciences, College of Science, Princess Nourah bint Abdulrahman University, P.O. Box 84428, 11671 Riyadh, Saudi Arabia; 5 Center of Research, Faculty of Engineering, Future University in Egypt, New Cairo, 11835 Egypt; 6 Department of Mathematics, College of Science, Qassim University, 51452 Buraydah, Saudi Arabia; 7 Department of Mechanical Engineering, College of Engineering in Wadi Alddawasir, Prince Sattam bin Abdulaziz University, Al-Kharj, Saudi Arabia; 8 Production Engineering and Mechanical Design Department, Faculty of Engineering, Mansoura University, Mansoura, P.O 35516 Egypt

Correction to: *Scientific Reports* 10.1038/s41598-023-40040-9, published online 07 August 2023

The original Article contains errors. Due to an error during formulation and simplification of equations (5) and (6), the gravitational force was retained. This term has now been removed.

The updated Equation (5) and Equation (6) take the form.

5 $$\left(u\frac{\partial u}{\partial x} + v \frac{\partial u}{\partial y}+w \frac{\partial u}{\partial z}-2\Omega v\right)=\frac{{\mu}_{hnf}}{{\rho}_{hnf}}\left(\frac{{\partial }^{2}u}{{\partial x}^{2}}+\frac{{\partial }^{2}u}{{\partial y}^{2}}+\frac{{\partial }^{2}u}{{\partial z}^{2}}\right)-\frac{{\mu}_{hnf}}{{\rho}_{hnf} }\frac{u}{{k}_{o}}$$

6 $$\left(u\frac{\partial v}{\partial x} + v \frac{\partial v}{\partial y}+w \frac{\partial v}{\partial z}-2\Omega u\right)=\frac{{\mu}_{hnf}}{{\rho}_{hnf}}\left(\frac{{\partial }^{2}v}{{\partial x}^{2}}+\frac{{\partial }^{2}v}{{\partial y}^{2}}+\frac{{\partial }^{2}v}{{\partial z}^{2}}\right)+-\frac{{\mu}_{hnf}}{{\rho}_{hnf} }\frac{v}{{k}_{o}},$$

Due to this correction, the thermal expansion coefficient (β) is now irrelevant, since that parameter only appears through the gravity-related term. The updated Equation (15) and Equation (16) take the form.

15 $${p}^{{\prime}{\prime}{\prime}}\left(\eta \right)=\frac{{\rho}_{hnf}}{{\rho}_{f}}\times \left\{{{p}{\prime}\left(\eta \right)}^{2}\left({p}{\prime}\left(\eta \right)+{q}{\prime}\left(\eta \right)\right)-2 \lambda \delta {q}{\prime}\left(\eta \right)+Z {p}{\prime}\left(\eta \right)\right\}\times {[1-\left({\phi}_{1}+{\phi}_{2}\right)]}^{5/2}$$

16 $${q}^{{\prime}{\prime}{\prime}}\left(\eta \right)=\frac{{\rho}_{hnf}}{{\rho}_{f}}\times \left\{{{q}{\prime}\left(\eta \right)}^{2}\left({p}{\prime}\left(\eta \right)+{q}{\prime}\left(\eta \right)\right)-2\frac{\lambda }{\delta } {p}{\prime}\left(\eta \right)+Z {q}{\prime}\left(\eta \right)\right\}\times {[1-\left({\phi}_{1}+{\phi}_{2}\right)]}^{5/2}$$

The updated Equation (18) takes the form.

18 $$\lambda =\frac{\Omega }{a},\delta =\frac{y}{x}, Z=\frac{{\mu}_{hnf}}{{a{\rho}_{hnf}k}_{o}}, Pr=\frac{{v}_{f}}{{k}_{f}}, \pi =\frac{4{\sigma }^{*} {{T}_{\infty }}^{3}}{{k}_{1} {k}_{f}}.$$

The updated Equation (28) and Equation (31) take the form.

28 $${{y}_{3}}{\prime}=\frac{{\rho}_{hnf}}{{\rho}_{f}}\times \left\{\left[{{y}_{2}}^{2}\times \left({y}_{1}+{y}_{4}\right)\right]-\left[2\times \lambda \times \delta \times {y}_{5}\right]+\left[Z\times {y}_{2}\right]\right\}\times {[1-\left({\phi}_{1}+{\phi}_{2}\right)]}^{5/2}$$

31 $${{y}_{6}}{\prime}=\frac{{\rho}_{hnf}}{{\rho}_{f}}\times \left\{\left[{{y}_{5}}^{2}\times \left({y}_{4}+{y}_{1}\right)\right]-\left[2\times \lambda \times \frac{1}{\delta }\times {y}_{2}\right]+\left[Z\times {y}_{5}\right]\right\}\times {[1-\left({\phi}_{1}+{\phi}_{2}\right)]}^{5/2}$$

Therefore, Tables 1 - 4 have been updated and numerical values in Table 6 and 7 have changed. The corrected tables are below as Tables 1, 2, 3, 4, 5, 6.

**Table 1:** Thermophysical properties of base fluid and nanoparticles.

Physical properties	Density (kg m^−3^)	Specific heat (J kg^−1^ K)	Thermal conductivity (W m^−1^ K)
Water	997	4179	0.614
Copper (*s*_1_)	8933	385	400
Aluminum oxide (*s*_2_)	3970	765	40
Titanium oxide (*s*_3_)	4250	686	8.96

**Table 2:** Thermophysical formulation for nanofluid (NF).

Properties	Nanofluid
Density	${\rho}_{nf}=(1-{(\phi }_{1}))\times {\rho}_{f}+{\phi}_{1}\times {\rho}_{s1},$
Dynamic viscosity	${\mu}_{nf}=\frac{{\mu}_{f}}{{[1-\left({\phi}_{1}\right)]}^{5/2}},$
Heat capacity	${\left(\rho {C}_{p}\right)}_{nf}=\left[1-\left({\phi}_{1}\right)\right]\times {\left(\rho {c}_{p}\right)}_{f}+{{\phi}_{1}\times (\rho {c}_{p})}_{s1},$
Thermal conductivity	$\frac{{k}_{nf}}{{k}_{f}}=\frac{{k}_{s1}+2{k}_{f}-2{\phi}_{1}({k}_{f}-{k}_{s1})}{{k}_{s1}+2{k}_{f}+{\phi}_{1}\times ({k}_{f}-{k}_{s1})},$

**Table 3:** Thermophysical formulation for hybrid nanofluid.

Properties	Hybrid Nanofluid
Density	${\rho}_{hnf}=\left[1-{(\phi }_{2})\right]\times \left[1-{(\phi }_{1})\times {\rho}_{f}+{\phi}_{1}\times {\rho}_{s1}\right]+ {\phi}_{2}\times {\rho}_{s2},$
Dynamic viscosity	${\mu}_{hnf}=\frac{{\mu}_{f}}{{[1-\left({\phi}_{1}+{\phi}_{2}\right)]}^{5/2}},$
Heat capacity	${\left(\rho {C}_{p}\right)}_{hnf}=\left[1-{(\phi }_{2})\right]\times \left[1-{(\phi }_{1})\times {\left(\rho {C}_{p}\right)}_{f}+{\phi}_{1}\times {\left(\rho {C}_{p}\right)}_{s1}\right]+ {\phi}_{2},$
Thermal conductivity	$\frac{{k}_{hnf}}{{k}_{nf}}=\frac{{k}_{s2}+2\times {k}_{nf}-2\times {\phi}_{2}\times \left({k}_{nf}-{k}_{s2}\right)}{{k}_{s2}+2\times {k}_{nf}+{\phi}_{2}\times \left({k}_{nf}-{k}_{s2}\right)},$ Here $\frac{{k}_{nf}}{{k}_{f}}=\frac{{k}_{s1}+2\times {k}_{f}-2\times {\phi}_{1}\times ({k}_{f}-{k}_{s1})}{{k}_{s1}+2\times {k}_{f}+{\phi}_{1}\times ({k}_{f}-{k}_{s1})}$

**Table 4:** Thermophysical formulation for trihybrid nanofluid.

Properties	Hybrid nanofluid
Density	${\rho}_{thnf}=\left[1-{(\phi }_{1})\right]\times \left[\left[1-{(\phi }_{2})\right]\times \left\{\left[1-{(\phi }_{3})\right]+{\phi}_{3}\times {\rho}_{s3}\right\}+ {\phi}_{2}\times {\rho}_{s2}\right]+ {\phi}_{1}\times {\rho}_{s1},$
Dynamic viscosity	${\mu}_{hnf}=\frac{{\mu}_{f}}{{[1-\left({\phi}_{1}+{\phi}_{2}+{\phi}_{3}\right)]}^{5/2}},$
Heat capacity	${\left(\rho {C}_{p}\right)}_{thnf}=\left[1-{(\phi }_{1})\right]\times \left[\left[1-{(\phi }_{2})\right]\times \left\{\left[1-{(\phi }_{3})\right]+{\phi}_{3}\times {\left(\rho {C}_{p}\right)}_{s3}\right\}+ {\phi}_{2}\times {\left(\rho {C}_{p}\right)}_{s2}\right]+ {\phi}_{1}\times {\left(\rho {C}_{p}\right)}_{s1},$
Thermal conductivity	$\frac{{k}_{thnf}}{{k}_{hnf}}=\frac{{k}_{s1}+2\times {k}_{hnf}-2\times {\phi}_{1}\times \left({k}_{hnf}-{k}_{s1}\right)}{{k}_{s1}+2\times {k}_{hnf}+{\phi}_{1}\times \left({k}_{hnf}-{k}_{s1}\right)},$ $Here \frac{{k}_{hnf}}{{k}_{nf}}=\frac{{k}_{s2}+2\times {k}_{nf}-2\times {\phi}_{2}\times \left({k}_{nf}-{k}_{s2}\right)}{{k}_{s2}+2\times {k}_{nf}+{\phi}_{2}\times \left({k}_{nf}-{k}_{s2}\right)},$ $and\,\frac{{k}_{nf}}{{k}_{f}}=\frac{{k}_{s3}+2\times {k}_{f}-2\times {\phi}_{3}\times ({k}_{f}-{k}_{s3})}{{k}_{s3}+2\times {k}_{f}+{\phi}_{3}\times ({k}_{f}-{k}_{s3})}$

**Table 5 (corrected table 6):** Numerical results of different parameters for skin frictions along *x*-*axis* $C{f}_{x}$ and *y*-*axis* $C{f}_{y}$ respectively.

$\lambda$	Z	$\gamma$	$\pi$	$Pr$	$C{f}_{x}$ for **NF**	$C{f}_{y}$ for **NF**	$C{f}_{x}$ for **HNF**	$C{f}_{y}$ for **HNF**	$C{f}_{x}$ for **THNF**	$C{f}_{y}$ for **THNF**
0.0	0.5	0.8	0.5	6.2	-1.71761	-0.78478	-1.42578	-0.651773	-0.590065	-0.270646
1.0					-1.1483	-2.03674	-0.953222	-1.69072	-0.394563	-0.699831
2.0					-1.02828	-2.98303	-0.853588	-2.47624	-0.353283	-1.02488
0.7	0.5	0.8	0.5	6.2	-1.23184	-1.68788	-1.02257	-1.40115	-0.423177	-0.579995
	0.8				-1.3843	-1.75916	-1.14912	-1.46031	-0.475577	-0.604422
	1.0				-1.48087	-1.80698	-1.22928	-1.5001	-0.508764	-0.620835
0.7	0.5	0.9	0.5	6.2	-1.17418	-1.81982	-0.974701	-1.51067	-0.403372	-0.625282
		1.0			-1.11793	-1.95966	-0.928001	-1.62675	-0.384054	-0.673279
		1.1			-1.06331	-2.10886	-0.882655	-1.75059	-0.365301	-0.724486
0.7	0.5	0.8	0.8	6.2	-1.23184	-1.68788	-1.02257	-1.40115	-0.423177	-0.579995
			1.0		-1.23184	-1.68788	-1.02257	-1.40115	-0.423177	-0.579995
			1.2		-1.23184	-1.68788	-1.02257	-1.40115	-0.423177	-0.579995
0.7	0.5	0.8	0.5	6.2	-1.23184	-1.68788	-1.02257	-1.40115	-0.423177	-0.579995
				7.2	-1.23184	-1.68788	-1.02257	-1.40115	-0.423177	-0.579995
				8.2	-1.23184	-1.68788	-1.02257	-1.40115	-0.423177	-0.579995

**Table 6 (corrected table 7):** Numerical outcomes of different parameters for Nusselt number ${Nu}_{x}$.

$\lambda$	Z	$\gamma$	$\pi$	$pr$	${Nu}_{x}$ for **NF**	${Nu}_{x}$ for **HNF**	${Nu}_{x}$ for **THNF**
0.0	0.5	0.8	0.5	6.2	5.5908	6.75477	9.26894
1.0					5.42556	6.55254	9.03166
2.0					5.20018	6.27624	8.71097
0.7	0.5	0.8	0.5	6.2	5.49293	6.63506	9.12841
	0.8				5.45173	6.58477	9.06730
	1.0				5.42488	6.55199	9.02749
0.7	0.5	0.9	0.5	6.2	5.67671	6.85755	9.42730
		1.0			5.85241	7.07021	9.71362
		1.1			6.02068	7.273849	9.98835
0.7	0.5	0.8	0.8	6.2	9.26570	10.0315	12.1943
			1.0		9.85894	10.5458	12.6331
			1.2		10.4210	11.0379	13.0583
0.7	0.5	0.8	0.5	6.2	5.49293	6.63506	9.12841
				7.2	5.96682	7.20818	9.90657
				8.2	6.40854	7.74239	10.6320

